# Animal models of congenital zika syndrome provide mechanistic insight into viral pathogenesis during pregnancy

**DOI:** 10.1371/journal.pntd.0008707

**Published:** 2020-10-22

**Authors:** Harish Narasimhan, Anna Chudnovets, Irina Burd, Andrew Pekosz, Sabra L. Klein

**Affiliations:** 1 W. Harry Feinstone Department of Molecular Microbiology and Immunology, Johns Hopkins Bloomberg School of Public Health, Baltimore, Maryland, United States of America; 2 Department of Gynecology and Obstetrics, Johns Hopkins School of Medicine, Baltimore, Maryland, United States of America; WRAIR, UNITED STATES

## Abstract

In utero Zika virus (ZIKV; family *Flaviviridae*) infection causes a distinct pattern of birth defects and disabilities in the developing fetus and neonate that has been termed congenital zika syndrome (CZS). Over 8,000 children were affected by the 2016 to 2017 ZIKV outbreak in the Americas, many of whom developed CZS as a result of in utero exposure. To date, there is no consensus about how ZIKV causes CZS; animal models, however, are providing mechanistic insights. Using nonhuman primates, immunocompromised mice, immunocompetent mice, and other animal models (e.g., pigs, sheep, guinea pigs, and hamsters), studies are showing that maternal immunological responses, placental infection and inflammation, as well as viral genetic factors play significant roles in predicting the downstream consequences of in utero ZIKV infection on the development of CZS in offspring. There are thousands of children suffering from adverse consequences of CZS. Therefore, the animal models developed to study ZIKV-induced adverse outcomes in offspring could provide mechanistic insights into how other viruses, including influenza and hepatitis C viruses, impact placental viability and fetal growth to cause long-term adverse outcomes in an effort to identify therapeutic treatments.

Zika virus (ZIKV) was first identified in the Ziika forest of Uganda in 1947 [1,2]. The virus caused isolated outbreaks in 2007 and 2013 in the Micronesia island of Yap and French Polynesia, respectively [3,4]. Those outbreaks caused little concern as they were associated mostly with asymptomatic infections and occasional mild-febrile symptoms, including fever, rash, myalgia, and conjunctivitis. In 2015, ZIKV became an international public health problem for pregnant women, in particular, as its association with developmental abnormalities in the neonates was established [1,3]. At the peak of the ZIKV epidemic in 2016 to 2017, numbers of ZIKV disease cases reached 216,207 in Brazil, and 8,604 neonates were born with malformations. In 2019, 1,649 probable cases of ZIKV infections were reported in pregnant women, 447 of which were confirmed by laboratory tests [3,5–8].

ZIKV is a single-stranded RNA virus, in the family *Flaviviridae*. Most ZIKV infections begin with the bite of a virus-infected *Aedes* mosquito. However, confirmed cases of nonvector infection, including sexual transmission, blood transfusion, and vertical transmission from mother to child, have been reported [8,9]. In this way, ZIKV is considered unique among the Flaviviruses, due to its ability to be sexually transmitted and teratogenic [1]. Following infection by mosquito bite, ZIKV targets the epidermis in addition to other cell types for replication [10]. Several receptors have been reported to mediate cellular attachment and entry, including Tyro3, AXL, and Mer (TAM) receptors, T cell immunoglobulin and mucin domain receptors, phosphatidylserine, C-type lectin receptors, and dendritic cell-specific intercellular adhesion molecule-3-grabbing non-integrin (DC-SIGN) [11]. Infection induces apoptosis of some cells via the p53 pathway, while other cells, including macrophages and monocytes, permit viral replication resulting in significant viremia [11]. Within the placenta, Hoffbauer cells (i.e., subtype of macrophages), cytotrophoblasts, and endothelial cells are permissive to productive infection [12,13]. Infection and replication of ZIKV within cells of the placenta gives ZIKV access to the fetal compartment where it exhibits a neurotropism, infecting neocortical neuroepithelial cells, neural stem cells (NSCs), and microglia [14–16]. ZIKV infection of the fetal brain leads to apoptosis, reduced cellular proliferation, and transcriptional alterations [17,18], which causes cortical alterations and neurodevelopmental anomalies.

The unique pattern of birth defects and disabilities caused by ZIKV infection during pregnancy has been termed congenital zika syndrome (CZS). Infants with CZS exhibit neuropathology and a clinical presentation similar to other common congenital infections, including *Toxoplasma gondii* or cytomegalovirus (CMV) infections. Certain distinct features of ZIKV pathogenesis have been exclusively observed in CZS: (1) severe microcephaly with partially collapsed skull; (2) thin cerebral cortices with subcortical calcifications; (3) macular scarring and focal pigmentary retinal mottling; (4) congenital contractures; and (5) marked early hypertonia and symptoms of extrapyramidal involvement [9]. Furthermore, CZS can cause malformations of brain structures, such as the hippocampus, corpus callosum, basal ganglia, thalamus, cerebellum, and brainstem [5,19]. The subcortical (as opposed to periventricular in CMV) calcifications are observed when the infection occurs later in pregnancy and the brain is macroscopically well formed. CZS is also associated with intrauterine growth restriction and less frequently pulmonary hypoplasia [9,20].

The extent and significance of long-term neurological sequelae of CZS is still unknown. The development of infants with CZS will most likely be severely impacted. Sensorineural hearing impairment, hypertonia, spasticity dysphagia, and tremors are residual long-term manifestations of CZS [21]. To determine the mechanisms driving these severe consequences of ZIKV infection in the fetus, research laboratories need animal models to study the pathogenesis of CZS [9,21]. In response to the ZIKV pandemic and the outbreak of the congenital syndrome, animal models were developed to study ZIKV pathogenesis and treatment approaches [13,22]. Many questions remain unknown about CZS and the maternal transmission, pathogenesis, clinical events, and the resulting neurological damage. In this review, we highlight the existing animal models of CZS, evaluate the current state of the field, and identify gaps in the literature that might help to find new directions to study CZS. We also illustrate how animal models of CZS could be used to mechanistically understand the broader implications of RNA viral pathogenesis during pregnancy, which has applicability to understanding the implications of other infections, such as influenza, hepatitis C, and severe acute respiratory syndrome coronavirus 2 (SARS-CoV-2) during pregnancy.

## Methods

We conducted a comprehensive search of peer-reviewed journal articles indexed in the National Library of Medicine’s MEDLINE and PUBMED database available as of March 31, 2020, using the following search terms: “Congenital Zika Syndrome,” “Zika virus and Pregnancy,” “Animal models of Zika,” and “Zika virus and Phylogenetic analysis.” While all studies modeling ZIKV pathogenesis and vertical transmission in pregnant animals were evaluated and reviewed, those exploring the use of vaccines and therapeutics to prevent viral replication were systematically excluded. Additionally, viral factors including strain/sequence differences across studies potentially affecting pathogenesis and disease phenotype were included.

### Nonhuman primates

Nonhuman primates (NHP) are an excellent model for CZS research, including neurophysiology, pathogenesis, and therapeutic development, largely due to their genetic closeness to humans (Table 1). ZIKV was first isolated from a sentinel rhesus macaque in 1947 at the Ziika forest, Uganda [2]. In the context of ZIKV, NHPs are a natural host and represent a major zoonotic reservoir. Similarities to humans, in the biology of pregnancy, including placental organization (haemomonochorial) and long gestation periods, make NHPs an attractive model to study the pathogenesis of CZS [23].

**Table 1:** Summary of NHP models of CZS.

NHP	Gestational day of inoculation	Virus strain (dose)	Route of inoculation	Viral detection	Phenotype	Reference
Olive baboon	102, 107, 101, 97	French Polynesia 2013 (H/PF/2013) (GenBank: KJ776791.2);10^4^ FFU– 1 mL	Subcutaneous	RT-qPCR, IHC	Neuroinflammation in fetus, viral RNA in fetal brain and placenta, inflammation in the placenta	[24]
Pigtail macaque	119	FSS13025, Cambodia 2010 (GenBank: KU955593); 5 × 10^7^ PFU	Subcutaneous	Plaque assay, RT-qPCR	Lag in the growth of biparietal diameter, enlarged ventricles in fetus	[26]
Pigtail macaque	82, 119; 60–63	FSS13025, Cambodia 2010 (GenBank: KU955593); 5 × 10^7^ PFU; Brazilian strain Fortaleza, 2015 (GenBank: KX811222); Mosquito salivary gland extract– 4 glands per inoculum)	Subcutaneous	Plaque assay, RT-qPCR	Gliosis of the fetal cortex, astrocytosis, decreased neural stem cells in fetal brain	[14]
Rhesus macaque	41, 50, 64, 90	Brazilian ZIKV SPH/2015 (GenBank: KU321639); (1 × 10^5^ PFU)	Intravenous and intraamniotic	Plaque assay, RT-qPCR, ISH	Fetal demise, decreased growth of the fetuses, brain calcifications, gliosis	[27]
Rhesus macaque	31, 38 103, 118	French Polynesia 2013 (H/PF/2013) (GenBank: KJ776791.2); 10^4^ PFU	Subcutaneous	Plaque assay, RT-qPCR, IHC	Small fetal head but no changes in the brain	[23]
Rhesus macaque	46	Puerto Rico 2015 PRVABC59 (GenBank: KU501215); 10^4^ PFU	Subcutaneous	Plaque assay, RT-qPCR, ISH	Fetal demise 49 days post infection, chorioamnionitis, placental infarcts and fetal ocular pathology	[28]
Rhesus macaque	31, 51, 114/115	Puerto Rico 2015 PRVABC59 (GenBank: KU501215); 10^5^ FFU	Subcutaneous	Plaque assay, RT-qPCR, IHC	Decreased oxygen permeability of the placental villi. Subtle abnormalities in fetal brain: thinner postcentral gyrus and missing secondary sulcus.	[100]
Rhesus macaque	42–49, 84–98	Brazil ZKV2015 (GenBank: KU497555.1); 10^3^ PFU	Subcutaneous	Plaque assay, RT-qPCR, IHC, ISH	Small fetal head, fetal demise. Fetal brain: decreased occipital gyrus, gliosis, hemorrhage	[30]
Rhesus macaque	First, second, and third trimesters (55dG, 110dG, and >)	Rio U-1/2016 (GenBank: KU926309); 10^4^ PFU	Subcutaneous	Plaque assay, RT-qPCR	Fetal demise (first trimester); Viral RNA in amniotic fluid (second trimester); No major effect (third trimester)	[101]
Marmoset	68, 79	Brazil ZIKV strain SPH2015 GenBank: (KU321639); 2.5 × 10^5^ PFU	Intramuscular	Plaque assay, RT-qPCR, IHC, ISH	Fetal demise and small fetal head	[30]
Olive baboon	86–95	Puerto Rico 2015 PRVABC59 (GenBank: KU501215); French Polynesia 2013 (H/PF/2013) (GenBank: KJ776791.2); 10^6^ FFU– 1 mL	Intravaginal	RT-qPCR, IHC	Vertical transmission observed only in PRABC59 inoculated animals. No adverse outcomes during study period.	[25]

CZS, congenital zika syndrome; FFU, focus forming unit; IHC, immunohistochemistry; ISH, in situ hybridization; NHP, nonhuman primate; PFU, plaque forming unit; RT-qPCR: real time-quantitative polymerase chain reaction; ZIKV, Zika virus.

The most utilized NHPs for the study of ZIKV pathogenesis during pregnancy include olive baboons (*Papio anubis*), pigtail macaques (*Macacca nemestrina*), rhesus macaques (*Macacca mulatta*), and marmosets (*Callithrix jacchus*) (Table 1). NHP models are superior because subcutaneous inoculation of ZIKV results in productive vertical transfer of virus from mother to offspring, consistent with humans. For example, subcutaneous infection of pregnant olive baboons (*P*. *anubis*) at various gestational ages, ranging from 97 to 107 days, results in mild to moderate rash and conjunctivitis in dams, with peak viremia at 5 to 7 days post inoculation (dpi) [24]. In addition to a systemic inflammatory response (i.e., broad induction of pro-inflammatory cytokines), ZIKV-specific immunoglobulin M (IgM) is generated by 14 dpi in the dams, with neutralizing immunoglobulin G (IgG) induced by 21 dpi [24]. Vertical transmission of ZIKV (French Polynesia 2013 –H/PF/2013) across the placenta to infect fetal tissue is only observed after 14 dpi, evidenced by the detection of ZIKV RNA. Following in utero infection, olive baboon fetuses exhibit neurological damage, with notable defects in radial glia, disorganized migration of neurons to cortical layers, and pathology in immature oligodendrocytes [24]. In addition to subcutaneous inoculation, intravaginal inoculation using ZIKV-infected olive baboon semen resulted in maternal viremia and vertical transfer, with the Puerto Rico (PRVABC59, 2015) strain being more virulent than the French Polynesia strain and causing more widespread dissemination to reproductive tissues and placenta [25].

In contrast to the olive baboons, pregnant pigtail macaques do not manifest any symptoms following subcutaneous infection at gestational day 119, although pregnant females develop ZIKV-specific IgG by 14 dpi [26]. Following subcutaneous infection of pregnant females with ZIKV (Cambodia 2010 –FSS13025), viral RNA is observed in placenta as well as fetal brain following delivery, but no infectious virus is detected, suggesting either resolution of infection or extremely low titers of replicating virus. Like in humans, imaging studies reveal an arrest of the fetal biparietal diameter reflecting an arrest in expansion of white matter in addition to axonal and ependymal injury [26], without resulting in microcephaly [14]. In pigtail macaques, subtle patterns are identified including periventricular T2-hyperintense foci, reduced noncortical brain volume as well as loss of neural progenitor cells in the subventricular zone and subgranular zone. Thus, clinically asymptomatic maternal ZIKV infection (at least with Cambodia 2010 –FSS13025) could result in fetal brain pathology, closely mimicking presentation in humans [26] and suggests that children exposed to ZIKV in utero may require long-term evaluation for possible neurodevelopmental defects irrespective of the presence/absence of microcephaly at birth.

Rhesus macaques are the most widely utilized NHP model to evaluate ZIKV pathogenesis in the context of CZS. Following intravenous or intraamniotic inoculation of the Brazil 2015 strain of ZIKV at gestation days 41 to 90 (i.e., corresponding to the first and second trimester of gestation), dams are viremic from 5 to 43 dpi, in the absence of developing clinical symptoms [27]. Despite having no clinical disease, ZIKV RNA traverses the placenta and is detected in fetal tissues, most notably the brain [27]. In neonates that survive in utero ZIKV infection, central nervous system (CNS) lesions, including calcification and loss of neural progenitor cells, are observed without displaying gross microcephaly [27]. Subcutaneous inoculation of rhesus macaques during the first trimester (gestation days 31 or 38) or late second/early third trimester (gestation days 103 or 118) results in detectable ZIKV RNA in fetal tissues, indicating successful vertical transmission with prominent neutrophilic infiltration at the maternal-fetal interface [23]. Additionally, infection with either Puerto Rico 2015 or French Polynesia 2013 strains of ZIKV during the first trimester, but not the third trimester, results in ocular pathology—inflammation of retina, choroid, and optic nerve of fetuses [23,28]. In rhesus macaques, ZIKV infection (Cambodia 2010/Brazil 2015) causes reduced oxygen permeability of the placental villi due to uterine vasculitis and villous damage [28]. Vascular insufficiency and neuroprogenitor cell dysfunction are hallmarks of CZS, resulting in fetal loss, smaller brain size, and histopathologic brain lesions [29]. In this model, the extent of fetal brain damage is more profound when ZIKV infection occurs early as opposed to later during pregnancy, thereby mimicking pathology in humans [29].

Even in marmosets, ZIKV pathogenesis during pregnancy results in neurological defects consistent with CZS. Intramuscular inoculation with the Brazil 2015 strain of ZIKV at gestation days 68 to 79 (i.e., period of placentation and embryonic neurodevelopment in marmosets) results in asymptomatic seroconversion and viremia in dams, with placental viral replication and cellular disorganization in the fetal brain [30]. Vertical transmission of ZIKV in pregnant NHP results in little to no disease in dams, but can cause fetal loss in rhesus macaque, olive baboon, and marmoset with ZIKV RNA detected in placenta, amniotic fluid, and various fetal tissues. ZIKV infection of pregnant rhesus macaques and marmosets also causes severe placental damage which may underlie development of symptoms consistent with CZS.

### Pigs

Parallels in brain development and growth between pigs and humans [31] have led to the study of flavivirus infections in domestic pigs [32]. Although organizational differences between the porcine and human placenta poses a challenge, the similarity of all major cell types within the placental–fetal mesenchyme suggests that ZIKV-induced pathology in the porcine placenta may be comparable to humans [33]. From the available literature, intraperitoneal or intravenous ZIKV inoculation (Puerto Rican strain–PRVABC59) of neonatal piglets only results in transient viremia, suggesting these conventional routes of inoculation would not result in successful vertical transmission [34]. Intrauterine inoculation results in infection of the placentas as well as fetal brains, with few piglets exhibiting microencephaly [34].

Inoculation of porcine conceptuses (fetus with fetal membrane) in utero with ZIKV (Puerto Rican strain–PRVABC59) at gestational day 50 causes persisted infection and detectable antibody responses in fetuses and dams [35]. Comparison of in utero infection with either Asian (PRVABC59) or African (DAK-AR-41524) ZIKV strains show that while both strains cause fetal infection, the African strain results in greater infection in utero [36] and elevated interferon alpha (IFNα) levels in otherwise healthy offspring, around 3 weeks after birth [37]. Moreover, in the absence of brain lesions, fetal brains exhibited profound transcriptional changes with male piglets specifically demonstrating more dramatic molecular pathology both within the brain and placenta. These findings are in agreement with a previous study describing higher risk of neurocognitive disorders in male mice following mild in utero ZIKV infection [38]. From these data, however, it does not appear that pigs are a superior model to evaluate either the mechanisms or therapeutic potential of treatments for CZS.

### Sheep

Sheep are a less recognized model of CZS, which may serve as a useful alternative to NHPs. In addition to being outbred and widely available to researchers, recent serological and in vitro studies indicate that sheep may be naturally exposed and susceptible to ZIKV infection [39,40]. Sheep have also been extensively used as a model for human pregnancy and fetal physiology, with studies of the maternal-fetal interface in sheep contributing to our understanding of fetal stress and intrauterine growth restriction [41]. Infection of pregnant sheep (gestational day 28–35) with an Asian strain (R103451) of ZIKV results in detectable levels of ZIKV RNA in maternal peripheral blood mononuclear cells (PBMCs) and seroconversion in the dams, in the absence of signs of sickness [42]. ZIKV infection during the first trimester also causes placental pathology, fetal growth restriction, and fetal loss [42]. Infection of pregnant sheep (gestational day 57 to 64) with a high dose of ZIKV (strain R103451, 10^8^ PFU) via the intravenous and subcutaneous route results in detectable infectious virus in the maternal spleen, placenta, and fetal organs including brain, spleen, and liver, with fetuses exhibiting significantly smaller head sizes as well as gross pathology following euthanasia by 15 dpi [43]. Taken together, these findings suggest that pregnant sheep are a useful large animal model to study ZIKV pathogenesis in the context of CZS.

### Mice

**Fig 1:**
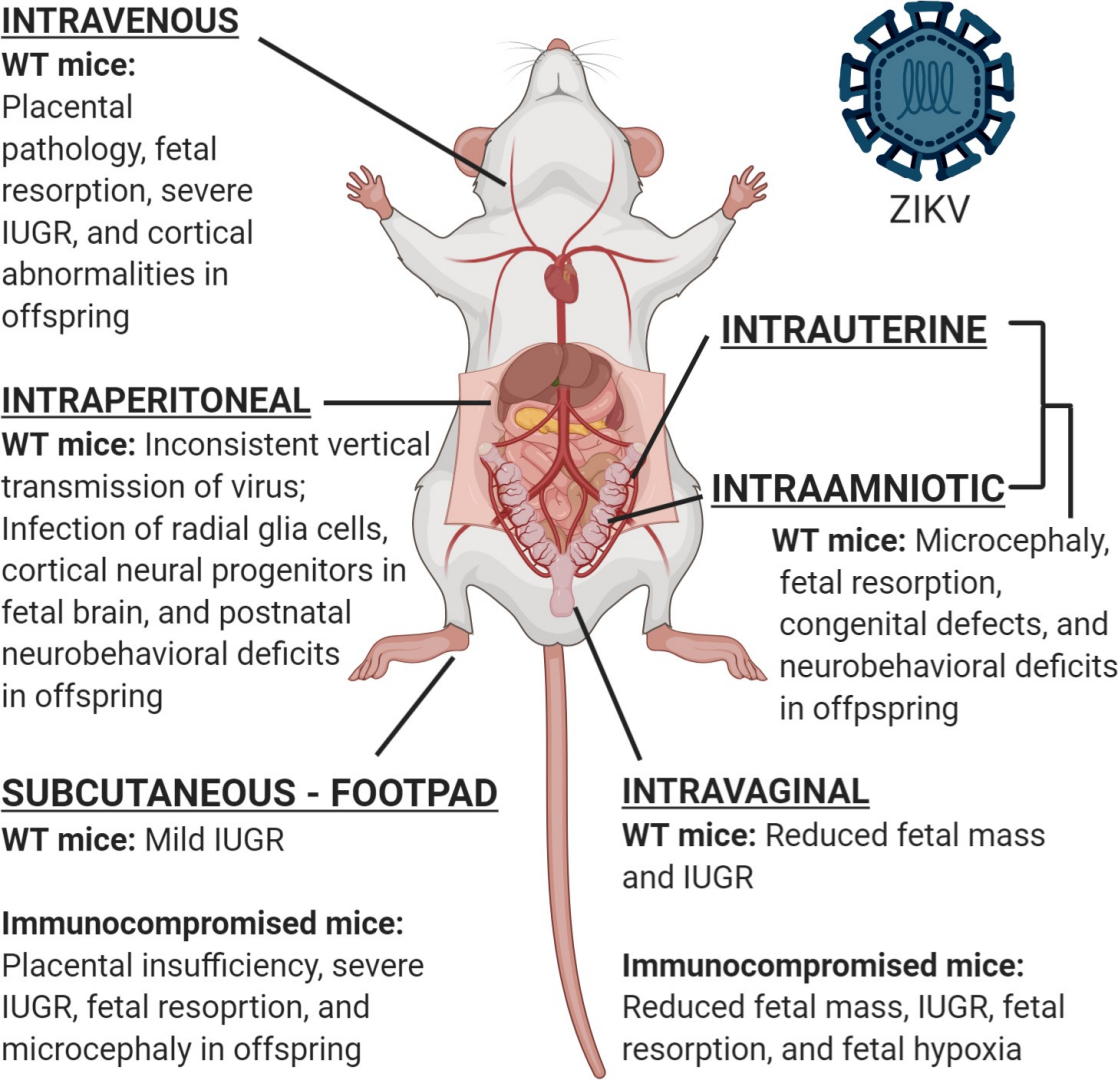
Routes of inoculation used in mouse models of CZS. Several routes of inoculation have been used to systematically evaluate the impact of historic and contemporary strains of ZIKV on fetal development, growth, and behavior, in either immunocompetent, WT, or immunocompromised pregnant mice. CZS, congenital zika syndrome; IUGR, intrauterine growth restriction; WT, wild-type; ZIKV, Zika virus. *Figure made using Biorender*.

Mice are a commonly used animal model to study viral pathogenesis [44]. In the context of pregnancy, the short breeding times and large litters are advantageous, while the heterogeneity in gestation periods and placental organization pose challenges to accurately model human pregnancy [33]. The lack of susceptibility of wild-type mice to various Asian and African strains of ZIKV further complicates disease modeling. The nonstructural (NS5) protein of ZIKV typically recognizes and degrades signal transducer and activator of transcription 2 (STAT2) in humans (and other susceptible hosts) to efficiently evade interferon (IFN) signaling and thereby replicate [45]. ZIKV NS5 is unable to bind murine STAT2, and the resulting lack of inhibition of IFN responses results in efficient control of viral replication and lack of overt disease [45]. To overcome this challenge, immunocompromised mice or modifications to the route of inoculation have been utilized to study the pathogenesis of ZIKV in mice with consideration of adverse fetal outcomes, including symptoms consistent with CZS (Table 2 and Fig 1).

**Table 2:** Summary of mouse models of CZS.

Mouse strain	Inoculation day	Virus strain and dose	Route of inoculation	Viral detection	Phenotype	Reference
Ifnar1^+/-^ dams (F) × C57BL/6 (M)	E6.5, E7.5	H/PF/2013 (GenBank: KJ776791.2); 10^3^ FFU– 50 uL	Subcutaneous	Plaque assay, RT-qPCR, IHC	Fetal resorption, IUGR, placental insufficiency	[49]
C57BL/6 (F) + MAR1-5A3 × C57BL/6 (M)	E6.5, E7.5	H/PF/2013 (GenBank: KJ776791.2); 10^3^ FFU– 50 uL	Subcutaneous	RT-qPCR	Mild IUGR, no fetal demise	[49]
Ifnar1^+/-^ dams (F) × C57BL/6 (M)	E6, E9, E12	Pariba 2015 (GenBank: KX280026); 10^3^ FFU– 50 uL	Subcutaneous	RT-qPCR	Placental insufficiency and fetal demise (E6); reduced cranial dimensions (E9)	[48]
C57BL/6 (F) + MAR1-5A3 × C57BL/6 (M)	E6, E9, E12	Pariba 2015 (GenBank: KX280026); 10^3^ FFU– 50 uL	Subcutaneous	RT-qPCR	ZIKV replication decreases with gestational age	[48]
Ifnar1^-/+^ (F) × C57BL/6 (M)	E7.5	PRVABC59 (GenBank: KU501215); DAKAR 41524 (GenBank: KX601166); 10^3^ PFU– 25 uL	Subcutaneous	Plaque assay, RT-qPCR	Fetal resorption, IUGR	[47]
Ifnar1^-/-^ (F) × Ifnar1^-/-^ (M)	E4.5, E8.5	FSS13025 (GenBank: KU955593); 2.5 × 10^4^ PFU to 5.2 × 10^5^ PFU– 10–20 μL	Intravaginal	Plaque assay, RT-qPCR, IHC	Fetal resorption (E4.5) and IUGR	[51]
IRF (Irf3^-/-^, Irf7^-/-^) KO (F) × IRF KO (M)	E4.5, E8.5	FSS13025 (GenBank: KU955593); 2.5 × 10^4^ PFU to 5.2 × 10^5^ PFU– 10–20 μL	Intravaginal	Plaque assay, RT-qPCR, IHC	Reduced fetal weight and high viral replication	[51]
Ifnar1^-/-^ (F) × C57BL/6 (M)	E5.5, E8.5	FSS13025 (GenBank: KU955593); 1.5 × 10^5^ PFU	Intravaginal	RT-qPCR, IHC	Fetal resorption (Ifnar1^-/+^ fetuses–E5.5), IUGR (more severe in Ifnar1^-/+^–E8.5), fetal hypoxia	[57]
SJL (F) × SJL (M)	E10–E13	Pariba 2015 (GenBank: KX280026); 1 × 10^3^, 4 × 10^10^ or 1 × 10^12^ PFU/mL– 200 uL	Intravenous	RT-qPCR	Severe IUGR, major cortical abnormalities in fetus	[5]
C57BL/6 (F) × C57BL/6 (M)	E9.5, E12.5	PRVABC59 (GenBank: KU501215); 3.4 × 10^5^ PFU– 100 uL	Intravenous (retro-orbital)	RT-qPCR, IHC	Placental pathology, fetal resorption, morphologically abnormal embryos and IUGR (primarily at E9.5)	[62]
CD-1 (F) × CD-1 (M)	E10, E14	Nigeria 1968 (IBH 30656) (GenBank: HQ234500); Puerto Rico 2015 (PRVABC59) (GenBank: KU501215); Cambodia 2010 (FSS13025) (GenBank: KU955593); Brazil 2015 (Pariba) (GenBank: KX280026); 10^6^ TCID_50_−100 uL	Intrauterine	TCID_50_ assay, RT-qPCR, IHC	Fetal resorption, placental dysfunction, reduced fetal cortical brain thickness	[13]
CD-1 (F) × CD-1 (M)	E10	Nigeria 1968 (IBH 30656) (GenBank: HQ234500); Puerto Rico 2015 (PRVABC59) (GenBank: KU501215); Brazil 2015 (Pariba) (GenBank: KX280026); 10^6^ TCID_50_−100 uL	Intrauterine	RT-qPCR, IHC	Fetal resorption, congenital malformations, neurocognitive defects in neonates	[63]
C57BL/6 (F) × C57BL/6 (M)	E4.5, E8.5	Cambodia 2010 (FSS13025) (GenBank: KU955593); 2.5 × 10^4^ PFU to 5.2 × 10^5^ PFU– 10–20 μL	Intravaginal	RT-qPCR, plaque assay, IHC	Reduced fetal weight, IUGR	[51]
FVB/NJ (F) × FVB/NJ (M)	E4.5	Brazil 2015 (HS-2015-BA-01) (GenBank: KX520666); 1 × 10^5^ PFU– 10 uL	Intravaginal	RT-qPCR	Reduced fetal weight, fetal resorption	[64]
C57BL/6 (F) × C57BL/6 (M)	E13.5	SZ01 2016 (GenBank: KU866423); 3 × 10^5^ PFU/mL– 300 uL (ip); 1.5 uL (LV)	Intraperitoneal, lateral ventricle of fetal brain	RT-qPCR, IHC	Radial glial cell infection in fetal brain, reduction in cortical neural progenitors	[67]
C57BL/6 (F) × C57BL/6 (M)	E15	SZ01 2016 (GenBank: KU866423); 200 PFU, 500 PFU– 100 uL	Intraamniotic	RT-qPCR, IHC	Reduction in fetal brain volume and cortical thickness, motor deficits, impaired vision	[68]
C57BL/6 (F) × C57BL/6 (M)	E13.5	ZIKA-SMGC-1 (GenBank KX266255); 1500 PFU/fetus	Intraamniotic	RT-qPCR, IHC	Low birth rate, microcephaly, eye abnormalities, paralysis	[69]
C57BL/6(F) × C57BL/6 (M)	E14.5	Mexico 2016 (MEX1-44) (GenBank: DQ859059); 1.7 × 10^6^ TCID_50_/ml approximately 1 μL	Lateral ventricle of fetal brain	RT-qPCR, IHC	Leaky fetal blood brain barrier, postnatal microcephaly, and brain damage	[70]
C57BL/6(F) × C57BL/6 (M)	E8.5	Puerto Rico 2015 (PRVABC59) (GenBank: KU501215); 10^4^ PFU	Intraperitoneal	Plaque assay, RT-qPCR, IHC	Neurobehavioral deficits	[15]
ICR (F) × ICR (M)	E13.5	SZ01 2016 (GenBank: KU866423); 6.5 × 10^5^ PFU/mL– 1 uL	Lateral ventricle of fetal brain	RT-qPCR, IHC	Dysregulation of NPC cell cycle, resulting in apoptosis and microcephaly	[18]
hSTAT2 KI (F) × hSTAT2 (M)	E6.5	Mouse-adapted Dakar 41525 (GenBank: MG758786); Dakar 41525 (GenBank: MG758785); 10^4^ to 10^6^ FFU PFU of ZIKV– 50 uL	Subcutaneous (footpad)	RT-qPCR	Viral RNA detected in fetuses	[22]
AG129 (F) × AG129 (M)	E7.5	Malaysia 1996 (P 6–740); 100 CCID_50_	Subcutaneous	RT-qPCR, TCID_50_, IHC	IUGR, fetal microcephaly, fetal resorption, transient hearing loss	[54]
Swiss-Webster + (F) × Swiss-Webster (M)	E4, E8, E12	HN16 (GenBank: KY328289.1); 1 × 10^4^ PFU– 10 uL	Subcutaneous	RT-qPCR	Fetal growth restriction (E4 and E8)	[53]
AG129 (F) × AG129 (M)	E3.5	Puerto Rico 2015 (PRVABC59) (GenBank: KU501215); 10^3^ PFU	Subcutaneous, intravaginal, sexual transmission from infected male	Plaque assay, RT-qPCR, IHC	Infectious virus detected in fetuses	[56]

CZS, congenital zika syndrome; FFU, focus forming unit; IHC, immunohistochemistry; IUGR, intrauterine growth restriction; NPC, neural progenitor cells; PFU, plaque forming unit; RT-qPCR, real time–quantitative polymerase chain reaction; TCID50, tissue culture infectious dose 50; ZIKV, Zika virus.

#### Immunocompromised mice

Prevention of IFN signaling either in transgenic type I IFN receptor knockout (KO) mice (Ifnar1^-/-^) or through the use of type I IFN receptor specific blocking antibody (MAR1-5A3) to treat wild-type (WT) C57BL/6 dams prior to inoculation with ZIKV has been employed to study adverse outcomes in offspring (Table 2) [36,46–52]. Because pregnant Ifnar1^-/-^ mice are highly susceptible to ZIKV, they can be productively infected with ZIKV (French Polynesia 2013 –H/PF/2013) via subcutaneous inoculation, which mimics mosquito bite challenge and results in successful infection of the placenta and fetal brains [49]. ZIKV-induced fetal brain damage and mortality are observed not only in homozygous but also in heterozygous (Ifnar1^-/+^) fetuses, suggesting that even a partial deficiency in type I IFN signaling is sufficient to induce fetal brain damage. Additionally, while infection (Brazilian strain–Pariba 2015) during early gestation (E6) results in placental insufficiency and fetal demise, infection during mid-gestation (E9) reduced cranial dimensions, and infection later in pregnancy (E12) causes no fetal disease [48], which has been replicated in other mouse models with other strains of ZIKV and routes of inoculation [13,53]. Using a subcutaneous route of inoculation, both Asian (Puerto Rico 2015 –PRVABC59) and African (DAK-AR-41524) ZIKVs result in adverse fetal outcomes, including brain damage [47,52]. A similar phenotype of microcephaly and fetal resorption was observed in AG129 (IFNα/β/γR KO), following subcutaneous inoculation of a Malaysian strain of ZIKV [54].

The ZIKV epidemic in the Americas revealed that the virus can be transmitted not only by mosquito vectors but also through sexual transmission [55]. Intravaginal inoculation of ZIKV (Cambodia 2010 –FSS13025) into pregnant *Ifnar1*^-/-^ mice at embryonic day 4.5 or 8.5 results in significant levels of viral RNA in the placenta and fetuses [51]. Intravaginal inoculation of ZIKV into pregnant IFN regulator factor (IRF) KO (*Irf3*^-/-^ *Irf7*^-/-^) mice causes even higher levels of viral RNA in the vagina and placenta compared to the *Ifnar1*^-/-^ dams. From these data, it is clear that inhibition of IFN signaling is necessary for productive systemic infection of mice with either Asian or African lineage strains of ZIKV. Dissemination of ZIKV (Puerto Rico 2015 –PRVABC59) to the fetus from IFN-deficient dams is more effective following sexual transmission from ZIKV-infected males than following either subcutaneous or intravaginal routes of inoculation [56].

The role of type I IFN signaling in modulating the development of disease during pregnancy can be investigated using transgenic mice, in which homozygous (*Ifnar1*^-/-^) females are mated with heterozygous (*Ifnar1*^-/+^) sires to generate a mixture of homozygous and heterozygous fetuses within the same uterine horns [57]. Virus (Cambodia 2010 –FSS13025) replicates to higher titers in the placenta of the homozygous *Ifnar1*^-/-^ than heterozygous *Ifnar1*^*-/+*^ following subcutaneous infection. Only the *Ifnar1*^-/+^ fetuses undergo resorption, whereas their *Ifnar1*^*-/-*^ littermates survive, in part because IFNAR signaling in the conceptus adversely affects the development of the placental labyrinth [57]. Whether IFNAR signaling in this model adversely affects brain development in the fetus has not been reported.

#### Immunocompetent mice

While earlier studies suggested that inoculation of ZIKV in immunocompetent mice results in minimal viral replication and no fetal defects, some studies revealed successful vertical transmission and adverse fetal outcomes [58,59]. Achieving productive infection of immunocompetent, WT mice requires using alternative strategies, such as higher titers of inoculum, unconventional routes of inoculation, or humanized mice (Table 2 and Fig 1). The first study demonstrating the ability of the Brazil strain of ZIKV (Pariba 2015) to induce birth defects was conducted using pregnant Swiss Jim Lambert (SJL) mice [60]. Intravenous infection at an extraordinarily high dose (200 uL– 10^10^ to 10^12^ PFU/mL) resulted in intrauterine growth restriction and microcephaly in the surviving pups, with significant amounts of viral RNA detected in their brains. SJL mice, however, have elevated levels of circulating T cells and are particularly susceptible to sarcomas and autoimmune conditions [61]. Within the SJL model, placental pathology rather than transmission of infectious virus is associated with adverse fetal outcome. Further, intravenous inoculation of an Asian ZIKV strain (Puerto Rico 2015 –PRVABC59) results in fetal abnormalities in the absence of vertical transmission of virus [62].

In an effort to mimic the heterogeneity observed in humans, outbred mice can be used, e.g., CD-1 mice, to study the transplacental transmission of several ZIKV strains of African and American/Asian lineages [13]. Using an intrauterine route of inoculation, in which the virus must still traverse the placental barrier to harm the fetus, infection with either African or Asian lineages of ZIKV results in greater levels of infection in the placenta and fetal head as well as fetal demise when infections occur at earlier (second trimester–E10) rather than later (third trimester–E14) gestational stages. In addition to productive ZIKV replication, second trimester ZIKV infection results in placental dysfunction and vascular damage, which can be blocked by coadministration of an interleukin 1 beta (IL-1β) receptor antagonist (IRA) suggesting that inflammation rather than virus replication causes placental and subsequent fetal damage [63].

Pregnant WT C57BL/6 dams are also susceptible to ZIKV (Cambodia 2010 –FSS13025) infection via intravaginal inoculation, with transmission of viral particles to the fetal brains, as detected by electron microscopy [51]. Although the infection of the WT dams only results in transient viremia followed by complete recovery, the embryos exhibit developmental defects, despite having undetectable infectious virus and viral RNA. Thus, it is possible that placental damage plays a more critical role in dictating fetal outcomes, rather than the presence of infectious virus in the fetus. Intravaginal inoculation (Brazil 2015) also resulted in the induction of several inflammatory genes as well as chemokines (CCL2 and CXCL10), which have been associated with microcephaly in humans [64]. A humanized STAT2 mouse strain also exhibited vertical transmission of the virus following subcutaneous inoculation at E6.5 with ZIKV (Dak-41525 and mouse-adapted Dakar) [65]. Although high concentrations of viral RNA were detected in both placentas and fetal heads, fetal mortality and phenotype were not characterized.

ZIKVs exhibit neurotropism due to the high expression of AXL protein, a cell surface receptor implicated in viral entry [66]. During embryogenesis, the virus targets multipotent neural progenitor cells to induce apoptosis of infected cells, resulting in microcephaly [18]. Several models have been developed to study the resulting neurological damage in the fetus following in utero infection. Fetal brain development is affected by ZIKV (strain SZ01 2016) infection of radial glial cells within the dorsal ventricular zones, the primary neural progenitor cells responsible for cortex development [67–69]. Impaired retinal development and motor defects are observed in pups born to dams infected with ZIKV via the intraamniotic route [68,69]. Extensive neuronal death, axonal rarefaction, and a leaky blood brain barrier are also observed in embryonic brains, along with the induction of several inflammatory genes [70]. Following intrauterine inoculation, ZIKV (Nigeria 1968 –IbH 30656) localizes in trophoblasts and endothelial cells in the placenta and in endothelial cells, microglial cells, and neural progenitor cells in the fetal brain [13].

To model postnatal developmental CZS in WT mice, intraperitoneal inoculation infected pregnant WT C57BL/6 dams with ZIKV (Puerto Rico 2015 –PRVABC59) and reduced body mass and head sizes in pups at postnatal day (PND) 12, with postnatal motor and memory impairments persisting into adulthood [15]. Following in utero infection of CD-1 mice with a Brazil strain (Pariba 2015) at E10, pups exhibited gross developmental abnormalities at birth (PND 0), including limb contractures, congenital syndactyly, and kinked tails [63]. Furthermore, intrauterine infection induced cortical brain injury in the pups, evidenced by reduced cortical thickness and neuroinflammation, due to increased activation of microglia [13,63]. As a result, pups develop both cognitive and motor defects by PND 5, which were ameliorated by reducing microglial activation via maternal administration of an IRA [63]. Immunocompetent mouse models are proving to be superior models for uncovering the mechanisms of CZS and suggest that placental inflammation in addition to virus replication is involved.

## Other rodents

### Rats

Rats have been used to study CZS as they naturally exhibit an immunocompromised phenotype during pregnancy, particularly during late gestation, similar to humans [71]. Following subcutaneous ZIKV inoculation, pregnant rats do not developed signs of sickness, but the pups show reduced hippocampal and cortical volumes with preliminary evidence of cerebro-cortical dysplasia [72]. However, as the presence of live, replicating virus was not confirmed within the dams or pups, whether these effects were directly mediated by the virus or by secondary mechanisms like activation of the maternal immune response remains unknown.

### Guinea pigs

Early attempts to model early life ZIKV infection in guinea pigs via intracranial inoculation of the Africa MR766 viral strain resulted in no apparent infection [73]. Extensive passaging of this strain through suckling mouse brains may have potentially compromised the tropism of the virus for use in other rodent species. Several subsequent studies, however, have demonstrated neurotropism and the ability of ZIKV to cause infection in adult guinea pigs through subcutaneous inoculation [74]. The ZIKV (French Polynesia 2013 –H/PF/2013) challenge of pregnant guinea pigs via subcutaneous route at 18–21 days of gestation results in no viral RNA detected in serum or tissues and no significant changes in pup size, despite detectable anti-ZIKV antibody in dams and pups [75]. Although guinea pigs may be useful to model the immune response to ZIKV, the virus itself may not be pathogenic enough to induce disease in pregnant immunocompetent guinea pigs and their offspring.

### Hamsters

In STAT2 KO hamsters, following infection with a Malaysia strain (P 6–740) of ZIKV via the subcutaneous route at 8.5 days post coitus results in productive infection of placental tissue and vertical transmission of infectious virus into the fetal brains [76]. In contrast, infection of WT pregnant dams does not result in detectable infectious virus or ZIKV RNA in fetal brains or placentas or affect pup body size as compared to control pups [49].

### Chicken embryos

Chicken embryos lack the majority of immunological mechanisms (e.g., antibody production, IFN signaling only functional after 8 days) typically associated with protection and recovery from viral infections [77]. In the context of CZS, chick embryos have proven extremely useful to characterize virus-induced brain damage and CNS abnormalities via imaging studies of embryos. The first study using chick embryos was conducted in 1952; embryos were inoculated with ZIKV via yolk, amniotic, or allantoic sac injections, which resulted in persistent infections for 9 dpi [78]. ZIKV replicated to equivalent titers in the brain and body of the inoculated chick embryos, resulting in ZIKV being regarded to be pantropic in this model. The strain of ZIKV used in this study, MR766, was passaged extensively in mouse brains, which may have potentially affected the tropism of the virus. More recently, using an intraamniotic inoculation of an Asian ZIKV strain into embryos at E2.5 and E5, using results in ventriculomegaly, stunted growth of the CNS and gross microcephaly [79]. Comparison of lineage-specific differences using 2 African (Nigeria IbH 30656 and Uganda MR766) and Asian (Mexico MEX1-44 and Brazil SPH 2015) isolates reveals that embryos infected with Asian strains as compared to African strains have a higher probability of survival [80]. Finally, cell tropism studies within the brain following ZIKV (French Polynesia 2013 –H/PF/2013) inoculation into the midbrain ventricle of E2 chicken embryos reveals nonuniform periventricular infection and a tropism for certain neuromeres (signaling centers), resulting in dysfunctional neural patterning [81]. While not a typical model, chicken embryos can be used to evaluate the neural implications of early life exposure to ZIKV.

### Viral determinants of disease

When ZIKV was discovered in 1947, the virus was primarily associated with minor outbreaks of self-limiting febrile illness. In 2013, epidemics in the Pacific Islands and the Americas began to include more severe clinical complications such as CZS and Guillain–Barre syndrome [1,3]. These clinical observations could be explained by the large number of cases that occurred after 2013, allowing for the detection of clinical complications that only penetrate in a limited number of infected individuals or they could represent new clinical features mediated by post-2013 ZIKV strains. Either of these hypotheses could be explained by mutations in the viral genome that altered virus infection efficiency or disease potential.

Phylogenetic analyses indicate the presence of 2 major geographically distinct genotypes—the African lineage and Asian lineage [3,82]. The most recent common ancestor of the African ZIKV is thought to have emerged around 1889, with the Asian ZIKV emerging between 1946–1966 [83]. The first documented migration of ZIKV outside of Africa and Asia, however, was to Yap Island in 2007, followed by its introduction to French Polynesia in 2013 and the Americas in 2015. All 2015 ZIKV isolates associated with the human epidemic clustered closely within the Asian lineage, forming an American clade with highest sequence identity to the French Polynesian isolate H/PF/2013, suggesting that the virus spreading through the Americas was imported from French Polynesia [82].

ZIKV has accumulated numerous mutations both prior to as well as during the outbreak in the Americas [84]. Whether any of these mutations have altered virus fitness for improved replication, infection, or disease potential still remains an open question. A more complete understanding of the viral genetic determinants that control these factors would improve our understanding of disease mechanisms as well as provide genetic signatures associated with increased disease potential, including the development of CZS in children.

The coding sequences of the African and Asian lineages have revealed approximately 75–100 amino acid changes across the 3,424 amino acids that comprise the viral genome [83]. Two substitutions in domain III of the E protein are hypothesized to alter receptor binding efficiency, while 2 within the E protein stem and transmembrane regions have been implicated in virion assembly and membrane fusion, respectively [85]. The E protein also harbors a prominent glycosylation site at the N154 position, which is lacking in several African lineage isolates [86]. Interestingly, this N-linked glycosylation site is considered to be essential for the infectivity and assembly of other flaviviruses such as Dengue and West Nile Virus [87]. Although several groups have reported differences between ZIKV strains and lineages in infectivity as well as phenotype of animals following infection, the molecular basis of these differences still remains poorly characterized [36,88,89]. Some studies suggest greater infectivity and more severe microcephaly following infection with Asian strains [52], while others report greater virulence of African strains [88,89]. To date, it has not been definitively shown that contemporary Asian lineage strains are more likely to cause microcephaly and other features of CZS than African lineage strains.

Phylogenetic and comparative amino acid sequence analyses revealed several conserved changes between isolates from the Yap Island and French Polynesia–D683E (E protein receptor), V763M/T777M (E protein), and S139N (prM protein) [83]. The S139N substitution in particular precisely coincides with the emergence of neurological disease in the epidemic [83]. Latin American isolates from 2015 also possessed an additional M/T2634V (NS5 protein), which may explain the increased incidence of microcephaly compared to the outbreak in French Polynesia [83]. However, it is important to note that these analyses are purely association studies based on epidemiological observation and thus require validation using animal disease models.

Experimental evidence demonstrated that the prM S139N substitution in recent Asian strains, previously associated with enhanced ZIKV infectivity and microcephaly [52], is not critical for adverse fetal outcomes [47]. Although the emergence of this substitution correlates with reports of microcephaly as well as other neurological abnormalities including Guillain-Barre syndrome [90], African strains (low passage) of ZIKV were also shown to be capable of causing fetal abnormalities [13,47]. Furthermore, reverse substitution of N139S in Asian strains did not alter infectivity or rates of resorption, suggesting that the S139 substitution is not principally required for fetal harm, at least in a mouse model [47]. Thus, CZS might not be a novel syndrome caused by a new ZIKV variant, but rather a preexisting disease that was recognized due to the large-scale outbreak in South America [50].

The effect of ZIKV adaptation must also be considered when assessing the pathogenesis and virulence of ZIKV in hosts [91]. The National Institutes of Health (NIH) reference strain (Uganda MR766) is among the most widely used strains in experimental studies. This strain, however, was serially passaged over 100 times in mouse brains, presumably leading to multiple mutations in the viral gene, which facilitate replication in mouse brain tissue [92]. Another African strain of ZIKV (Nigeria 1968 –IbH 30656) has also been passaged in suckling mouse brains over 21 times [92]. Unfortunately, the changes in virus sequences as a consequence of passage history have not been documented, to date. The development of a mouse-adapted strain of ZIKV Dakar 41525 (Senegal 1984) by passaging the WT virus in Rag1^-/-^ mice has provided some details about ZIKV mutations that enable the virus to adapt to mice [22]. Specifically, a mutation in the nonstructural protein 4B (NS4B) (G18R) facilitates replication in mouse brains as well as increased pathogenicity. ZIKV NS4B also diminishes innate immune responses by modulating the activation of TANK-binding kinase 1 (TBK1), resulting in reduced IFN-stimulated gene expression as well as IFN-β production [17]. Analyses of neurocognitive outcomes in mouse pups following in utero exposure to mouse-adapted Dakar will be required to better model CZS in an animal model.

## Conclusions and future directions

As a result of the ZIKV outbreak that occurred in 2016 to 2017, numerous models were developed to characterize and mechanistically study how ZIKV causes CZS in offspring (Box 1). The 2 most common animal models include NHPs and mice. Vertical transmission of ZIKV in pregnant NHPs is very effective in rhesus macaques, marmosets, and baboons. Adverse fetal outcomes including fetal demise are observed in all NHP models, with viral RNA detected in the placenta, amniotic fluid, and several fetal tissues, including the brain. Studies of NHP raise the possibility that CZS is caused by significant placental pathology and induction of a type I/II IFN response, which should be explored in future studies.

**Box 1: Key learning points**

- Nonhuman primates, as natural hosts, exhibit vertical transmission of ZIKV and effectively mimic fetal abnormalities that occur during CZS in humans ranging from fetal demise to subtle brain pathology.
- Mice require genetic/chemical manipulations to induce an immunocompromised state or atypical routes of inoculation, in the context of wild-type immunocompetent mice, to permit vertical transmission of ZIKV. A gestational stage effect was observed in several studies, with worse fetal outcomes following infection at earlier gestational stages, similar to humans.
- Mutations within the viral genome as well as the effect of adaptation to host organisms in causing CZS requires further investigation.
- Other RNA viruses, including those that can be transmitted to the fetus (e.g., hepatitis C virus) and those that are not vertically transmitted (e.g., influenza viruses), cause adverse fetal outcomes.

Both immunocompetent and immunocompromised mice have been successfully utilized to define the immunological and viral determinants of adverse fetal outcomes following ZIKV infection (Box 1). Mice also have been integral for testing a variety of genetic and chemical manipulations as well as unconventional routes of inoculation. Studies in mice have demonstrated a correlation between gestational timing of infection and severity of fetal and neonatal outcomes. Although mice are not natural hosts of ZIKV, they are a valuable resource to study mechanistic aspects of CZS. Future studies should further characterize the role of the placental immune response in causing characteristics of CZS in mice.

With the knowledge that pregnant women, unborn fetuses, and neonates represent 3 populations of high-risk individuals for severe complications from viral infection, we need greater insights into the unique mechanisms of adverse disease outcomes (Box 2). Generally, infectious pathogens during pregnancy can be divided into 3 broad categories—maternal, neonatal, or congenital infections, based on the pathogenesis and disease outcome [93]. ZIKV causes fetal or congenital infection, which is characterized by mild or no disease in pregnant females, but occasional vertical transmission and severe congenital disease in the fetus; thus, the focus of modeling is to uncover how to mitigate vertical transmission and adverse fetal outcomes.

**Box 2: Five key papers in the field**

- Miner JJ, Cao B, Govero J, Smith AM, Fernandez E, Cabrera OH, et al. Zika Virus Infection during Pregnancy in Mice Causes Placental Damage and Fetal Demise. Cell. 2016;165(5):1081–91.
- Vermillion MS, Lei J, Shabi Y, Baxter VK, Crilly NP, McLane M, et al. Intrauterine Zika virus infection of pregnant immunocompetent mice models transplacental transmission and adverse perinatal outcomes. Nat Commun. 2017;8:14575.
- Adams Waldorf KM, Stencel-Baerenwald JE, Kapur RP, Studholme C, Boldenow E, Vornhagen J, et al. Fetal brain lesions after subcutaneous inoculation of Zika virus in a pregnant nonhuman primate. Nature Medicine. 2016;22(11):1256–9.
- Cugola FR, Fernandes IR, Russo FB, Freitas BC, Dias JLM, Guimarães KP, et al. The Brazilian Zika virus strain causes birth defects in experimental models. Nature. 2016;534(7606):267–71.
- Wu K-Y, Zuo G-L, Li X-F, Ye Q, Deng Y-Q, Huang X-Y, et al. Vertical transmission of Zika virus targeting the radial glial cells affects cortex development of offspring mice. Cell Research. 2016;26(6):645–54.

Hepatitis C virus (HCV) is another RNA virus where 3%–10% of infected pregnant women will transmit the virus to the fetus. While approximately 25% of infected children ultimately clear the virus within 6 years of age, the risk of severe liver damage and/or failure due to chronic infection is prominent [94]. Similar to ZIKV, majority of infected women do not develop clinical manifestations during pregnancy [94]. There exist inconsistencies in literature regarding pregnancy outcomes with a large study indicating the risk of preterm delivery, low birth weight, and congenital abnormalities in infants born to HCV-infected mothers [95], while others suggest HCV infection is not a risk factor for adverse fetal outcomes [96]. There is, however, a correlation between maternal viral load and vertical transmission [97], prompting the need for mechanistic animal studies to assess antiviral therapies that are safe and efficacious during pregnancy.

In contrast, other RNA viruses, such as influenza viruses, seem to cause maternal infection, with heightened disease severity in pregnant females, but with rare or inconsequential transmission and disease in the fetus [98]. During the 2009 H1N1 (swine flu) pandemic, over 5% of total fatalities were pregnant women, even though they only constituted only 1% of the total population [98]. Seasonal epidemics show a similar trend, with women in the third trimester of pregnancy being over 3 times more likely to die from influenza-related illnesses [99]. While fetal demise and preterm delivery have been observed, lack of vertical transmission suggests indirect effects of infection on fetal outcomes [98,99]. Preclinical animal studies as well as clinical studies of pregnant women are needed to accurately determine the immunological phenotype, both systemically and in the placenta, mediating the increased severity of influenza infection in pregnant individuals. Animal studies modeling viral pathogenesis during pregnancy are necessary for appropriate development and testing of prophylactic and therapeutic treatments of viral infection during pregnancy and early neonatal life.
